# Continuous Objective Assessment of Near Work

**DOI:** 10.1038/s41598-019-43408-y

**Published:** 2019-05-06

**Authors:** Rachel Williams, Suyash Bakshi, Edwin J. Ostrin, Lisa A. Ostrin

**Affiliations:** 1 College of Optometry, University of Houston, 4901 Calhoun Rd, Houston, TX 77004 USA; 2 Computer Science, University of Houston, 4901 Calhoun Rd, Houston, TX 77004 USA; 3 MD Anderson Cancer Center, University of Texas, 1515 Holcombe Blvd, Houston, TX 77030 USA

**Keywords:** Biotechnology, Risk factors

## Abstract

Evidence regarding the role of near work in myopia is conflicting. We developed the RangeLife, a device for continuous, objective measurement of working distance. Four devices were built, calibrated, and validated. Then, adult subjects wore the device on weekdays and weekend days, while simultaneously wearing an actigraph device for objective measurements of light exposure and activity. Subjects maintained an activity log and answered a visual activity questionnaire. RangeLife data were downloaded and binned into 0.10 m intervals. Objective diopter hours (dh), a weighted measure of near work, were calculated. Diopter hours for all subjects were significantly higher on weekdays (14.73 ± 4.67 dh) compared to weekends (11.90 ± 4.84 dh, p = 0.05). 94 ± 1.85% of near and intermediate viewing distances were recorded when the subjects were exposed to mesopic and indoor photopic light levels (<1000 lux), and 80.03 ± 2.11% during periods of sedentary physical activity (<320 counts per minute). Subjective reports of time viewing near and intermediate distances significantly overestimated objective measures (p = 0.002). The RangeLife was shown to provide reliable measures of viewing distance, and can be further utilized to understand potential influences of viewing behaviors on refractive error.

## Introduction

Refractive development is regulated by a complex interaction between genetic, environmental, and behavioral factors^1,2^. Potential influences include time outdoors^3–6^, near work^7,8^, physical activity^9,10^, education^8,11^, nutrition^12^ and urbanization^13^. Several studies have reported an association between myopia and increased time studying and reading^14–17^. Rose, *et al*., reported the highest odds ratio for myopia was for low time spent outdoors and a high time spent performing near work. However, results regarding the influence of near work have been conflicting, with some studies reporting no significant increased risk of myopia with increased near work^18–20^. Inconsistent results concerning the role of near work and myopia onset and progression are likely due to the variable and subjective nature by which near work has been assessed. Previous studies evaluating near work in children have traditionally been performed with parent questionnaires, which are subject to recall and parent biases^21,22^. In addition, surrogate measures are often used for near work. For example, some studies have used education, intelligence^23^, and occupation^24,25^ as surrogates for near work.

Besides parent questionnaires, other studies have utilized diaries to quantify near work^26^; however, diaries are limited by subject compliance. Another technique previously utilized is the experience sampling method^27–30^, in which a pager was dispensed to subjects to send alerts throughout the day. Following each page, subjects were asked to complete a self-report about activities at the moment of the page. While this technique provided real-time sampling of activities and was shown to be capable of detecting the proportion of time spent doing near work, only discreet time points were sampled, typically 5–6 times throughout each day, and results depended on the response rate of the subject, averaging approximately 81%^28^.

“Diopter hours” is a metric used to quantify near work behaviors, and can be calculated from visual activity questionnaires. Diopter hours weights various near activities and viewing distances to determine a comprehensive value of near work exposure^14,26^. Diopter hours represents a weighted sum of the time spent in particular activities according to the accommodative demand required to perform the task. For example, hours spent reading at a near distance is given more weight than hours spent using a computer at an intermediate distance. However, this metric does not fully describe the complexity of viewing behaviors that may influence eye growth, such as the temporal properties of near viewing. Relevant viewing behaviors that might affect eye growth include the duration of near viewing sessions, intermittent breaks during near viewing, and absolute viewing distance^31^. Recent studies in children have shown that continuous reading and closer reading distances are associated with myopia^16,32^. Studies in animals have shown that experimental myopia is dependent on the temporal pattern of defocus or form deprivation, rather than the total duration of the myopiagenic stimulus^33,34^. For example, Napper, *et al*., showed that brief interruptions of form deprivation with normal vision significantly decreased the magnitude of myopia in chicks^34^. These findings suggest that reading breaks might be beneficial during prolonged near tasks. The development of a device that provides continuous, objective assessment of viewing distance offers the opportunity to ultimately assess such behaviors to understand their influence on myopia onset and progression in humans.

Wearable electronic monitoring devices are being more commonly utilized to objectively and precisely quantify environmental and behavior factors^35^. Several studies have utilized light, activity, and sleep monitors to begin to understand the influence of these behaviors in refractive development. Recent studies have provided objective evidence that light exposure is related to time of year^36^, and that myopic children tend to spend less time outdoors than emmetropic children^4^. Importantly, these studies revealed discrepancies between traditional subjective reports and objective data for both adults and children^36,37^.

One continuously measuring range finding device has been previously utilized to assess reading distance in adult high and low/non-myopes^38^. The authors used ultrasonic technology mounted on a headband to continuously measure and log viewing distance while subjects read a newspaper for 10 minutes. The results showed that the device was repeatable with high sensitivity, and that high myopes had a shorter working distance than low/non-myopes. Additionally, the authors found that the subject’s self-reported reading distances varied greatly from objective measures. However, the device was not utilized in subjects’ habitual environment and was limited to two 10 minute experimental periods in the lab.

The development and implementation of wearable devices for continuous measurement of working distance can be used in conjunction with previously validated light exposure and activity monitors to provide a more complete assessment of visual activity relevant to eye growth and myopia development. Here, we employed infrared time-of-flight technology to develop a spectacle frame mounted device, the RangeLife, for continuous measurement of working distance. Our goals were to calibrate and validate the RangeLife device according to known distances and subjects’ activity logs, and to compare objective measures to traditional subjective visual activity questionnaires. Additional goals included assessing light exposure and activity levels during near viewing, and comparing near viewing behaviors in non-myopic and myopic young adults.

## Methods

### Instrumentation and calibration

All components for the RangeLife were acquired from Adafruit, USA. The RangeLife device consists of a time-of-flight distance sensor which uses a class 1 940 nm laser with nanosecond technology (VL53L0X). The time-of-flight principle is a method for measuring the distance between a sensor and an object, based on the time difference between the emission of a signal and its return to the sensor, after being reflected by an object, enabling accurate distance ranging regardless of the object’s surface characteristics. The sensors were controlled by an Arduino microprocessor coupled to a real-time clock (DS3231 Precision RTC) and a micro secure digital (SD) card data logger (Feather M0 Adalogger). Technical specifications for the distance sensor can be found at https://www.adafruit.com/product/3317. Components were connected using the built-in Arduino Inter-Integrated Circuit (I2C) bus. Power was supplied by a rechargeable 2500 mAh lithium polymer battery to provide approximately 16 hours of continuous distance measurements. The sensor is 1 × 1 × 0.2 cm and attaches to the user’s glasses, with a 1 m wire leading to the microprocessor which is housed with the RTC and battery in a plastic enclosure with a belt clip. The device was programmed to collect data continuously at 1 Hz and write onto files on the SD card. Open-source programming libraries for the sensor, clock, and data logger components were used to write running code for the device using the Arduino language. All code and schematics are available on request.

Four devices were constructed and used in this study. Each device was tested on one occasion by one observer for known distances from 0.05 to 2.0 m in 0.05 m intervals to assess stability of the measurements and generate a calibration function. For each device, the sensor was placed along a millimeter scale parallel to ground, and directed towards a wall. The device was set to begin recording at 1 Hz, and started at a position 0.05 m from the wall. Every 150 seconds, the device was moved away from the wall by 0.05 m. The recording continued until the device reached 2.0 m. The data were downloaded, and the distances measured by the device were plotted against actual distances from the millimeter scale. Data from all four devices were plotted and linear regression analysis was used to determine a calibration function.

Beam diameter refers to the width of the incident laser beam that bounces off of an object. The more narrow the beam diameter, the more precision the device will have to detect small objects in the line of sight. The device was directed towards a wall and projected onto a concentric millimeter scale. The beam diameter was determined for each of the four devices, for distances from 0.1 to 0.5 m in 0.1 m intervals. The observer viewed the beam through an infrared viewer to measure beam diameter at each distance. For 0.6 m and greater, the projected beam on the surface was too faint to detect with the infrared viewer; therefore, beam diameter was interpolated from 0.6 to 1 m. A linear regression was fit to the data, and the beam diameter was calculated in degrees.

### Subject testing

Subject testing was conducted with the four RangeLife devices described above. Healthy adult subjects ages 22–41 (n = 23) participated in this study. The study was approved by the Committee for Protection of Human Subjects at the University of Houston and was in accordance with the tenets of the Declaration of Helsinki. Subjects provided informed consent after learning the details of the study. All subjects had best corrected visual acuity of 20/20 or better and no ocular disease.

Subjects answered a custom activity questionnaire, the University of Houston Near work, Environment, Activity, and Refraction (UH NEAR) survey (see Appendix 1), adapted from previously published visual activity surveys^39^. Refractive status (myopic or non-myopic) was determined by self-report from the questionnaire using an indirect method technique, which has been shown to have reasonable sensitivity and specificity for determining whether a subject is myopic^40^. Additionally, subjects were drawn from the student and staff population of the University of Houston College of Optometry and were confident in reporting their refractive status. Previously utilized visual activity questionnaires do not take into account the abundant use of hand held devices, such as smartphones and tablets, that has become more prevalent in the past decade. Therefore, we created the UH NEAR survey, adapted not only from near activity questionnaires, such as the Sydney Myopia Study^39^, but also from public health and psychology questionnaires. Items were created to account for electronic and printed viewing tasks separately, and reduce redundancy and uncertainty between items. For example, video games, an item commonly included in published questionnaires, creates ambiguous results because subjects engage in video games at near (hand held devices), intermediate (computer) and far (television) distances. We attempted to clarify these factors through the UH NEAR survey.

RangeLife devices were fully charged prior to wear, then mounted on the right temple of a spectacle frame directed approximately 4° nasally, so that the beam was aligned at midline at 0.40 m. Devices were mounted to subjects’ habitual spectacle frame, or, if the subject did not wear spectacles, on a provided frame with plano lenses. The wire ran along the right temple, behind the ear, and to an enclosure, measuring 9 × 6 × 4 cm, that held all of the components. The enclosure was attached via a belt clip or placed in the subject’s pocket.

Subjects were asked to wear the device from the time they woke up until going to bed, except for showering or swimming. Because the device was affixed to the temple of the spectacle frame, subjects were encouraged to turn their head rather than rotating their eyes to ensure the device was measuring along the line of sight. Subjects kept a detailed log of activities while the device was worn to compare objectively measured distances to corresponding activities. Additionally, subjects wore an actigraph and light sensor device (Actiwatch Spectrum, Respironics, Phillips, OR, USA) while wearing the RangeLife so that patterns of light exposure and activity could be correlated to viewing distance. Light exposure (lux) and physical activity (counts per minute) were averaged over one minute epochs, as previously described^36^. Time outdoors was defined as minutes per day exposed to greater than 1000 lux^4^. Following each full day of wear, RangeLife data were downloaded and binned into 0.10 m intervals to calculate the number of minutes per day spent viewing distances from 0.10 to 1.0 m. Distances were categorized from very near viewing (0.1 cm) to “far” viewing (>1.0 m, Table 1).

**Table 1:** Viewing distance classifications from 0.1 to >1.0 m with example activities for each category.

Range	Viewing Distance	Classification	Example activities
Near	0.1 to <0.2 m	Extremely near	Hand held devices
	0.2 to <0.3 m	Very near	Hand held devices, printed material
	0.3 to <0.4 m	Fairly near	Printed material
	0.4 to <0.5 m	Near	Printed material, computer monitor
	0.5 to <0.6 m	Moderately near	Computer monitor
Intermediate	0.6 to <0.7 m	Near intermediate	Computer monitor
	0.7 to <0.8 m	Intermediate	Conversing with others
	0.8 to <0.9 m	Moderately intermediate	Conversing with others
	0.9 to <1.0 m	Far intermediate	Conversing with others, cooking
Far	≥1.0 m	Far	Television viewing, driving, outdoor activity

### Data analysis

Data are presented as mean ± standard error unless otherwise noted. Calibration and beam diameter data were fit with linear regressions. Subjective diopter hours (dh) were calculated from the questionnaire using Eq. 1, and objective diopter hours were calculated from RangeLife data using Eq. 2. Both subjective and objective diopter hours were calculated separately for weekdays and for weekends, then mean daily diopter hours were calculated using Eq. 3. Weekday versus weekend data, and subjective versus objective data for each subject were compared using paired two-tailed t-tests. Data for refractive error groups were compared using unpaired two-tailed t-tests with equal variance. Alpha level <0.05 was considered significant.

1 $$\begin{array}{rcl}{\rm{Subjective}}\,{\rm{Diopter}}\,{\rm{Hours}} & = & [3\times ({\rm{hours}}\,{\rm{reading}}\,{\rm{print}}\\  &  & +\,{\rm{hours}}\,{\rm{drawing}},\,{\rm{painting}}\,{\rm{writing}}\\  &  & +\,{\rm{hours}}\,{\rm{using}}\,{\rm{hand}}-{\rm{held}}\,{\rm{devices}})]\\  &  & +\,[2\times ({\rm{hours}}\,{\rm{using}}\,{\rm{computers}}\\  &  & +\,{\rm{playing}}\,{\rm{board}}\,{\rm{games}}\,{\rm{or}}\,{\rm{cards}})]\\  &  & +[1\times ({\rm{hours}}\,{\rm{watching}}\,{\rm{tv}})]\end{array}$$

2 $$\begin{array}{rcl}{\rm{Objective}}\,{\rm{Diopter}}\,{\rm{Hours}} & = & (3\times {\rm{hours}}\,{\rm{viewing}}\,0.1\,{\rm{m}}\,{\rm{to}}\, < 0.5\,{\rm{m}})\\  &  & +\,(2\times {\rm{hours}}\,{\rm{viewing}}\,0.5\,{\rm{m}}\,{\rm{to}}\, < 0.8\,{\rm{m}})\\  &  & +\,(1\times {\rm{hours}}\,{\rm{viewing}}\,0.8\,{\rm{m}}\,{\rm{to}}\, < 100\,{\rm{m}})\end{array}$$

3 $${\rm{Daily}}\,{\rm{diopter}}\,{\rm{hours}}=[({\rm{weekday}}\,{\rm{diopter}}\,{\rm{hours}}\times 5)+({\rm{weekend}}\,{\rm{diopter}}\,{\rm{hours}}\times 2)]/7$$

## Results

### Instrumentation

**Figure 1:**
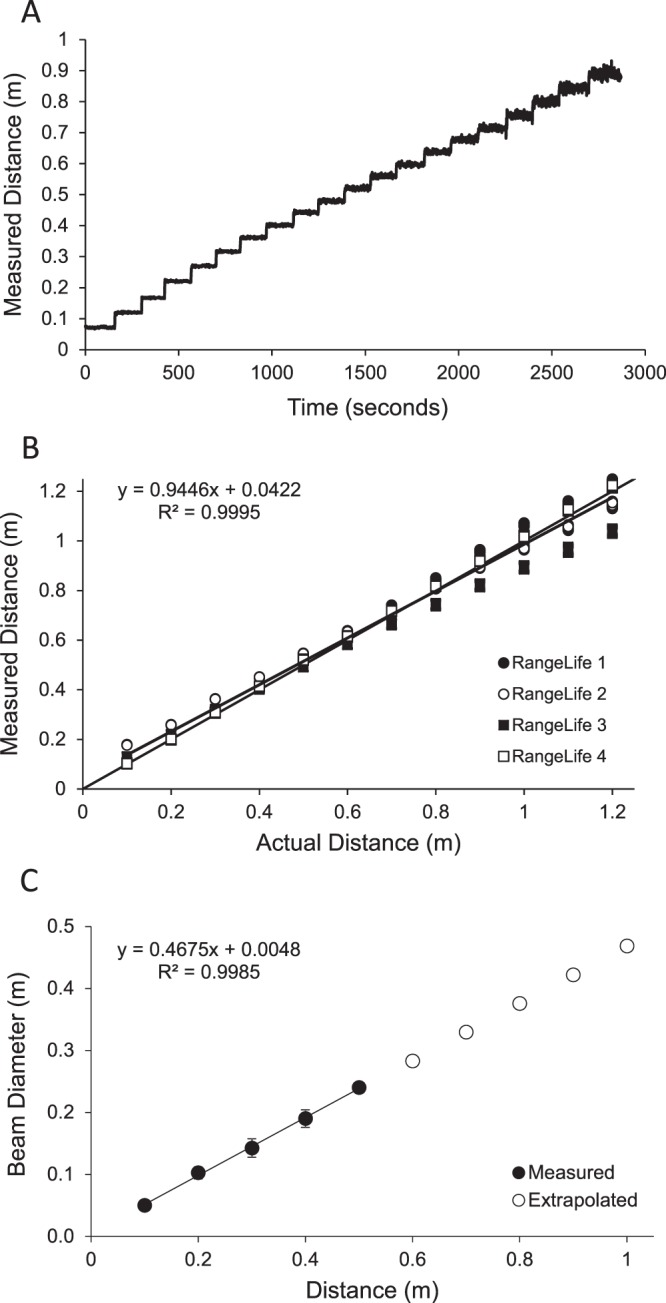
(**A**) Representative example of raw data from one RangeLife device as it is moved away from a wall from 0.05 to 1.0 m in 0.05 m steps approximately every 150 seconds. (**B**) Linear regression (solid line) showing the relationship between actual distances versus device-measured distances for each of the four devices. Dashed line represents the 1:1 line. (**C**) Infrared beam diameter with distance from a surface. Solid symbols are measured values and open symbols are extrapolated from the linear regression (solid line). Error bars represent standard deviation for the four devices.

During calibration testing, each of the four devices showed high stability with no noise in the recording. Raw data from one representative device are shown in Fig. 1A. The devices demonstrated a linear relationship with measured distances from 0.10 to 1.2 m. For the mean of all four devices, the regression equation was y = 0.9446x + 0.422 (Fig. 1B). Beyond 1.2 m, distances were registered by the devices as “out of range”. For all remaining analyses, distances from 0.1 to <0.6 m were considered “near”, from 0.6 to <1.0 m were considered to “intermediate”, and ≥1.0 m were considered “far.” The standard deviation of the measurement at 0.10 m was ±0.002 m, at 0.40 m was ±0.003 m, and at 1.0 m was ±0.012 m. The infrared beam diameter ranged from 0.05 m (at 0.1 m distance) to 0.47 m (at 1.0 m distance). The beam diameter increased linearly with distance (y = 0.4675x + 0.0048, Fig. 1C), which represents a beam width of 27°.

### Subjects

Mean subject age was 27.7 ± 5.6 years, with a range of 22–41 (10 female, 13 male). Thirteen subjects were myopic and 10 subjects were non-myopic, with no significant difference in age between refractive error groups (p = 0.17).

### Subjective data

One subject with mean daily diopter hours of 63.57 dh was identified as an outlier (Tukey outlier test) and not included in data analysis. Subjective diopter hours for all other subjects was greater for weekdays (26.64 ± 9.16 dh) than for weekends (23.25 ± 10.75 dh, p = 0.02). Subjective daily diopter hours were not significantly different between refractive error groups for weekdays (p = 0.27) or weekends (p = 0.93).

### Objective data

**Figure 2:**
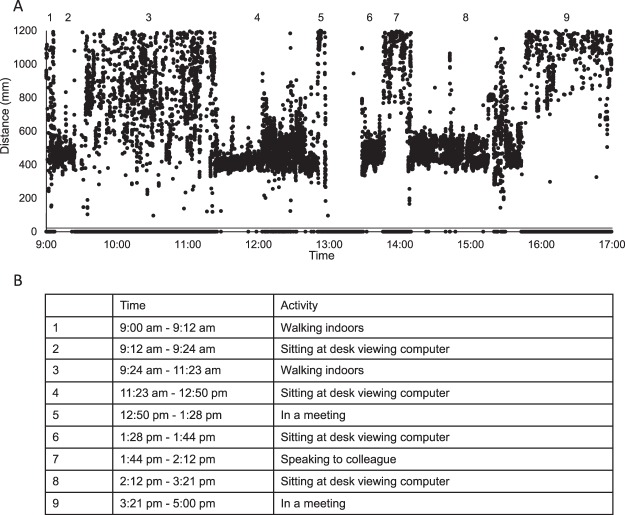
(**A**) Raw distance data collected at 1 Hz from the RangeLife for one representative subject on a weekday, 9:00 am to 5:00 pm. Gaps in the data represent “out-of-range” viewing, >1200 mm. Numbers 1–9 represent activities from the subject’s activity log, and are listed in panel (B).

For objective measures from the RangeLife device, 19 subjects wore the device for two days each, one weekday and one weekend day. Three subjects wore the device for one weekday only, and one subject wore the device for one weekend day only. On average, subjects wore the device for 12.7 ± 2.2 hours on the weekday and 12.4 ± 1.7 hours on the weekend day (p = 0.61). Based on all subjects’ activity logs and objectively measured distance data, activities were correlated to measured distances at corresponding times. Examples activities for each distance are provided in Table 1. Raw data from the RangeLife for one subject for a weekday, 9:00 am to 5:00 pm, are shown in Fig. 2A, and correlating activities from the subject’s activity log are provided in Fig. 2B.

Comparisons between objectively measured weekday versus weekend hours were calculated for the 19 subjects who wore the device on both days. Objective diopter hours was greater for weekdays (14.73 ± 4.67 dh) than for weekends (11.91 ± 4.82 dh, p = 0.05). When analyzed by refractive status, objective weekday diopter hours for non-myopic subjects was 12.73 ± 4.99 dh, which was not significantly different than weekend diopter hours, 11.81 ± 3.7 dh (p = 0.34). For myopic subjects, objective weekday diopter hours was 16.4 ± 3.52 dh, which was also not significantly different than weekend diopter hours, 11.97 ± 5.53 (p = 0.10). While myopic subjects tended to have greater diopter hours during the week than non-myopic subjects (16.4 vs 11.97 dh, respectively), the differences did not reach statistical significance (p = 0.06). When weekday and weekend objective diopter hours were weighted for the week (Eq. 3), the mean daily objective diopters for non-myopes was 12.66 ± 5.15 dh, and for myopes was 14.98 ± 2.86 dh (p = 0.22).

**Figure 3:**
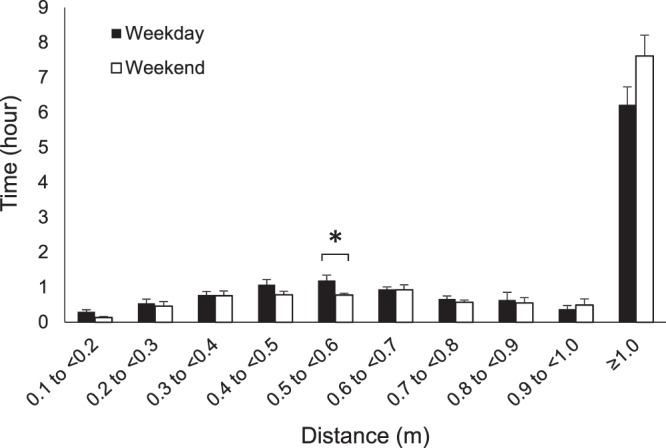
Objectively measured mean daily time (mean hours ± standard error) viewing distances from 0.1 to greater than 1.0 m for weekdays (solid bars) and weekends (open bars). Time spent viewing distances from 0.5 to <0.6 m was significantly greater on weekdays at p < 0.05, while differences in other binned distances did not reach statistical significance *p < 0.05.

Because there were no significant differences between refractive error groups, the remaining analyses were performed for non-myopic and myopic subjects together. Time spent viewing in each near to intermediate 0.1 m bin, as well as far viewing, for weekdays and weekends, is shown in Fig. 3. While there was a tendency for subjects to spend more time viewing near to intermediate distances and less time viewing distance on weekdays, the only 0.1 m interval in which the time was significantly different between days was for the 0.5–0.6 m range (p = 0.04).

**Figure 4:**
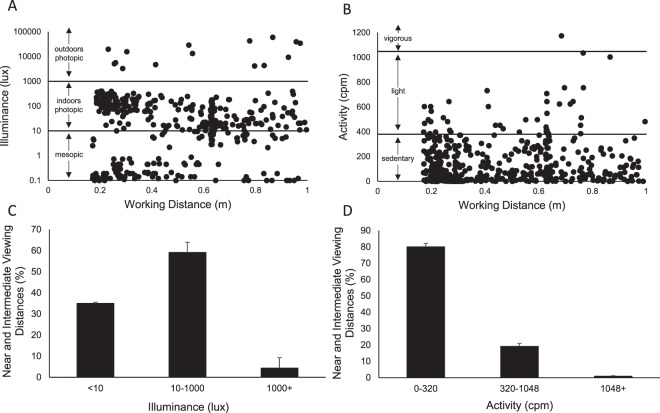
Objectively measured working distance (RangeLife) plotted with illuminance (lux) and activity counts per minute (cpm) as measured with the Actiwatch for a representative subject (**A,B**) and all subjects (**C**,**D**, mean ± standard error).

Near to intermediate working distances (0.1 to <1.0 m) were compared to objective measurements of light exposure and activity, as measured with the Actiwatch. Data for a representative subject are shown in Fig. 4A,B, and data for all subjects are shown in Fig. 4C,D. Subjects primarily engaged in near to intermediate working distances when they were exposed to indoor photopic luminance levels from 10 to 1000 lux, with a mean of 59.10 ± 4.78% of near to intermediate working distances detected in this light level range. Near to intermediate working distances were also detected when the subjects were exposed to mesopic luminance levels less than 10 lux, with a mean of 35.01 ± 4.94% of near to intermediate working distances detected in this light level range. There were minimal near to intermediate working distances measured when the subjects were exposed to outdoor illumination greater than 1000 lux (4.34 ± 1.27%). For physical activity, 80.03 ± 2.11% of near to intermediate working distances were measured while subjects demonstrated sedentary physical activity levels (<320 counts per minute)^41^, with 19.09 ± 1.95% of near to intermediate working distances measured while the subjects demonstrated light physical activity (320 to 1048 counts per minute). 0.88 ± 0.28% of near to intermediate working distances were detected when the subject was engaged in moderate or vigorous activity (>1048 counts per minute).

### Subjective vs objective data

**Figure 5:**
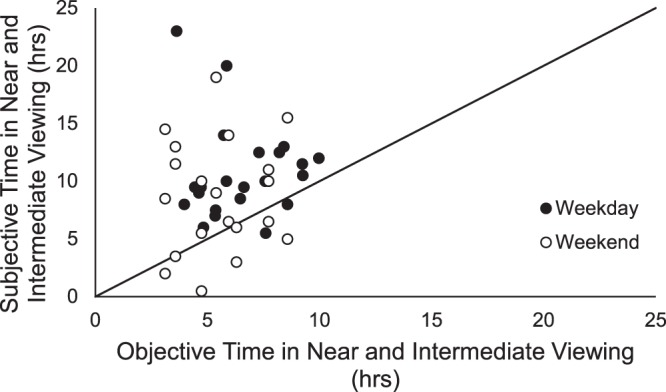
Objectively measured time spent viewing near to intermediate distances versus subjectively reported time for weekdays (solid symbols) and weekends (open symbols). Line represents 1:1 relationship.

From the questionnaire, time spent in near and intermediate work was determined by asking subjects to estimate the amount of hours spent viewing a computer, viewing a handheld device, reading printed material, drawing, painting or writing, and playing cards or board games. Subjects reported a mean daily average of 10.34 ± 0.85 hours engaged in near and intermediate work. From the RangeLife device, time spent in near and intermediate viewing was determined by summing the amount of time spent viewing distances from 0.1 to <1.0 m. Objectively measured mean daily time viewing near and intermediate distances was 6.25 ± 0.39 hours. Objective measures were significantly less than subjective reports (p = 0.002), and there were no correlations between subjective reports and objective measures for either weekdays (p = 0.78) or weekends (p = 0.76, Fig. 5).

Time spent outdoors was determined subjectively by asking the subjects to estimate the amount of time spent outdoors in physical activity, leisure activity, and driving or riding in a car. Subjective reports of time spent outdoors was 2.75 ± 0.30 hours per day. Objective measurement of time outdoors was determined from the Actiwatch by summing the number of minutes per day the subject was exposed to greater than 1000 lux, which was 1.44 ± 0.46 hours per day, and significantly less than subjectively reported time outdoors (p = 0.002).

## Discussion

The results of this study demonstrate the validity and feasibility of continuous, objective assessment of near and intermediate viewing distances using the RangeLife device. We have presented the first objective quantification of viewing distances throughout a day for young adults. We developed a classification scheme for categorizing near to intermediate distances in 0.1 m bins, and established a method for calculating objective diopter hours. These results support further use of the device to assess differences between larger groups of subjects of various ages and refractive status to understand the influence of near work and viewing behavior in myopia. In particular, the device can be used in future studies to assess not only working distance, but also temporal properties of viewing behaviors.

Here, near and intermediate viewing was assessed during weekdays and weekends to begin to understand how behaviors might vary based on school/work days versus leisure days. In young adult subjects, we found that objective diopter hours were greater for weekdays (14.73 dh) versus weekends (11.91 dh). While there was a trend for myopic subjects to spend more time engaged in near and intermediate viewing than non-myopic subjects, the difference did not reach statistical significance. We speculate that a larger sample size might clarify whether differences in viewing behaviors exists between refractive error groups in young adults. However, it is not unexpected that adult subjects with fairly homogenous occupations (i.e. students and staff at a university) would demonstrate similar behaviors. Saw, *et al*., reported that age of onset of myopia was related to close up work activity in early childhood, but not close up work as a young adult; subjects were all military conscripts^11^. Future studies aimed at assessing objectively measured near viewing patterns in younger subjects are imperative in determining the role of near viewing behaviors in refractive development.

By analyzing multiple objective measures of behavior, including working distance using the RangeLife and light exposure and physical activity using an Actiwatch, we can begin to tease apart specific behaviors that may influence eye growth. We found that the majority of near and intermediate work was carried out while the subjects were exposed to mesopic (<10 lux) and indoor photopic (<1000 lux) illuminance. Additionally, subjects were primarily engaged in a sedentary level of physical activity during near and intermediate viewing. Several studies have suggested that bright outdoor light exposure is protective for myopia development^5,42^; however, it remains unclear if viewing behaviors during bright light exposure, such as engaging in near work while outdoors, might contribute to myopia development. Future studies making use of objective measures for these multifactorial components will help to elucidate contributing behaviors to myopia, and may allow researchers and clinicians to suggest modifiable behaviors that will reduce the onset and progression of myopia.

In this study, subjective and objective diopter hours were calculated to determine a near work index using each method. Diopter hours weights various near activities and viewing distances to determine a comprehensive value of near work exposure. The diopter hours metric was introduced by Zadnik, *et al*.^43^, in 1994 as part of the ORINDA study, and has been utilized in several studies, albeit using different calculations^14,26^. For example, Zadnik, *et al*., determined diopter hours by adding together “three times the number of hours spent reading (for pleasure or studying), two times the number of hours spent playing video-type games, and the number of hours spent watching television,” and reported mean 53.8 ± 26.8 dh in eighth grade children^14^. Saw, *et al*., calculated diopter hours by multiplying various near work activities by the reciprocal of the distance for each activity, and reported ranges from 0.008 to 0.21^26^. Here, subjective diopter hours from the questionnaire averaged 25.67 ± 9.2 dh and objective hours from the RangeLife averaged 14.0 ± 4.0 dh. It is not unexpected that there would be discrepancies between subjective and objective diopter hours because of the variability in subjective data, and the differences in how subjective and objective diopter hours were calculated here. Therefore, the metric is best used as a relative measure to compare refractive error groups or across time periods when using a similar methodology.

Studies employing objective wearable sensors to assess reading distance^38^ and time outdoors^36,37^ have reported discrepancies between subjective reports and objective measures. We used the newly developed UH NEAR survey for subjective reports of visual activity. However, we still found variability between subjective and objective quantification of viewing distances, with most subjects overestimating time spent in near work as compared to objective measures derived from the RangeLife. These findings further justify implementation of objective measures to quantify environmental and behavioral factors. While objective measures are ideal for precisely quantifying visual activity, questionnaires will still be a beneficial supplement to objective measures to fully characterize behaviors.

We found that the RangeLife device was capable of logging continuous measures of viewing distance with high sample rate (1 Hz) and no noise in the data. Continuous measurements with a high sample rate will allow for a more precise quantification of viewing distance, which, in future studies, can be analyzed in terms of the duration of continued near work with quantification of near viewing breaks that may be interspersed within the near work task. The absence of noise in the data eliminates the need for filtering and potential loss of data. Another important feature of range sensing devices is that they have a narrow beam diameter and can detect when the line of sight is directed at small targets, such as hand-held electronic devices. The beam diameter of the RangeLife is narrow enough such that it was able to detect hand held devices, books, and computer monitors. These characteristics are ideal for assessing near viewing behaviors that have been linked to refractive error, including the duration and frequency of near work^16,44^, distance of reading material^38^, and other reading habits^45^.

The RangeLife device presented some limitations in measuring viewing distance. The reliable measurable range was limited to 0.1 to 1.0 m, and therefore, distances greater than 1.0 m could not be quantified and were classified as “far” for the purpose of this study. Another limitation was that the device was mounted to a spectacle frame and therefore not necessarily aligned with the line of sight, which may have resulted in some inaccuracies as subjects rotated their eyes. We attempted to minimize this confounding factor by encouraging subjects to turn their head towards targets rather than rotating their eyes. A previous study using a similar device tested the directional sensitivity and found that the device retained its sensitivity even when the target was at a substantial angle from the measuring beam^38^. Additionally, the authors found that the region of maximal sensitivity was about 30° for working distances of 40 to 100 cm. Therefore, small rotations from the line of sight are not expected to have a large impact on the measured viewing distance; however, future studies are aimed at quantifying the directional sensitivity for the RangeLife. Another limitation was that the device transmits data via a cord leading to an enclosure with a belt clip, which might present some difficulty with subjects who are involved in sports or other vigorous physical activity. Our adult subjects did not report problems in the current study, even during exercise. However, future instrument optimization is aimed at designing the components such that data can be stored within the sensor, eliminating the need for a wired enclosure.

In conclusion, the RangeLife was shown to provide continuous, objective, and reliable measures of near and intermediate viewing distances. Additionally, significant differences were demonstrated between RangeLife measures and traditional questionnaire data. The RangeLife device can be further implemented to assess viewing behaviors that may influence refractive error development in children and young adults.

## Supplementary information

Visual Activity Survey

## Data Availability

Data are available by request.
