# Supplementary material for: Continuous Objective Assessment of Near Work
Source: Sci Rep. 2019 May 6;9:6901. doi: 10.1038/s41598-019-43408-y (PMC6503122; doi:10.1038/s41598-019-43408-y)
Supplement: Supplementary file 1 — Visual Activity Survey [file 41598_2019_43408_MOESM1_ESM.pdf]

(please do NOT include your name on this questionnaire)

# Continuous Objective Assessment of Near Work

Rachel Williams,[1] Suyash Bakshi,[2] Edwin Ostrin,[3] Lisa Ostrin[1*]

1 College of Optometry, University of Houston, 4901 Calhoun Rd, Houston, TX 77004, USA

2 Computer Science, University of Houston, 4901 Calhoun Rd, Houston, TX 77004, USA

3 MD Anderson Cancer Center, University of Texas, 1515 Holcombe Blvd, Houston, TX 77030, USA

* corresponding author: lostrin@central.uh.edu

(please do NOT include your name on this questionnaire)

# UH NEAR Survey Adult

Age: ________________________________

Are you Hispanic or Latino? _____yes ______no

Race (select all that apply): _____white _____black/African American _____Native American

_____Asian _____Other: ____________________

In what city and state do you live? _______________________________________

Were you born in the United States? _____yes ______no

If no, what country were you born? ________________________________

What type of housing do you live? _____apartment _____single family house

What type of neighborhood do you live? _____urban _____rural

Do you wear glasses or contact lenses? _____yes ______no

If yes, is the correction for seeing: _____ distance ____near _______both ______unsure

Are your glasses bifocals? _____yes ______no ______unsure

If known, please provide your prescription: Right eye: ____________________________

Left eye: _____________________________

Age when started wearing glasses or contact: _______ years old

Have you undergone the following ocular procedures (select all that apply): _____cataract removal

_____ LASIK/laser refractive correction _______orthokeratology ______unsure ______none

What is your occupation? __________________________________________________________

**If possible, please ask your student clinician, doctor or optician today:**

**What is the patient's spectacle prescription from today's exam for whom this survey is filled out?**

**right eye** ________________________________________________________________________

**left eye** _________________________________________________________________________

(please do NOT include your name on this questionnaire)

**Please mark the number of hours per day you spend involved in these activities:**

| | Weekday | | | | | | Weekend Day | | | | | |
|---|---|---|---|---|---|---|---|---|---|---|---|---|
| | Not at all | Less than 1 hour | 1-2 hours | 3-4 hours | 5-6 hours | 7 or more hours | Not at all | Less than 1 hour | 1-2 hours | 3-4 hours | 5-6 hours | 7 or more hours |
| 1. Outdoor physical activities (sports, hiking, walking, biking, running) | ☐ | ☐ | ☐ | ☐ | ☐ | ☐ | ☐ | ☐ | ☐ | ☐ | ☐ | ☐ |
| 2. Outdoor leisure activities (eating, sitting or resting, outdoors) | ☐ | ☐ | ☐ | ☐ | ☐ | ☐ | ☐ | ☐ | ☐ | ☐ | ☐ | ☐ |
| 3. Daytime driving or riding in a vehicle (car, bus or train) | ☐ | ☐ | ☐ | ☐ | ☐ | ☐ | ☐ | ☐ | ☐ | ☐ | ☐ | ☐ |
| 4. Indoor physical activities (exercise, sports, martial arts) | ☐ | ☐ | ☐ | ☐ | ☐ | ☐ | ☐ | ☐ | ☐ | ☐ | ☐ | ☐ |
| 6. Viewing a TV screen (movies, video games) | ☐ | ☐ | ☐ | ☐ | ☐ | ☐ | ☐ | ☐ | ☐ | ☐ | ☐ | ☐ |
| 7. Viewing a computer screen (work, browsing, computer games) | ☐ | ☐ | ☐ | ☐ | ☐ | ☐ | ☐ | ☐ | ☐ | ☐ | ☐ | ☐ |
| 8. Viewing a handheld electronic device (smart phone, tablet, handheld video games, kindle) | ☐ | ☐ | ☐ | ☐ | ☐ | ☐ | ☐ | ☐ | ☐ | ☐ | ☐ | ☐ |
| 9. Reading printed material (newspaper, magazines, books, work) | ☐ | ☐ | ☐ | ☐ | ☐ | ☐ | ☐ | ☐ | ☐ | ☐ | ☐ | ☐ |
| 11. Drawing, painting, or writing | ☐ | ☐ | ☐ | ☐ | ☐ | ☐ | ☐ | ☐ | ☐ | ☐ | ☐ | ☐ |
| 14. Playing card or board games (not electronic) | ☐ | ☐ | ☐ | ☐ | ☐ | ☐ | ☐ | ☐ | ☐ | ☐ | ☐ | ☐ |

15. How many hours of sleep to you get on a week night? ___________________ weekend night? __________________
